# No dislocations after primary hip arthroplasty with the dual mobility cup in displaced femoral neck fracture in patients with dementia. A one-year follow-up in 20 patients

**DOI:** 10.1051/sicotj/2016050

**Published:** 2017-02-10

**Authors:** Anders Elneff Graversen, Stig Storgaard Jakobsen, Pia Kjær Kristensen, Theis Muncholm Thillemann

**Affiliations:** 1 Department of Orthopedic Surgery, Hospital Horsens 8700 Horsens Denmark; 2 Department of Orthopedic Surgery, Aarhus University Hospital 8000 Aarhus Denmark

**Keywords:** Arthroplasty, Dementia, Dislocations, Dual mobility cup, Femoral neck fracture

## Abstract

*Purpose*: The aim of this study was to describe the dislocation rates, reoperation rates and mortality 30 day and one year following THA with AVANTAGE^®^ dual mobility cup among dementia patients with an acute displaced intracapsular femoral neck fracture.

*Patients and methods*: From 2010 to 2014 we identified 20 hip fracture patients with dementia, who have had total hip arthroplasty with the AVANTAGE^®^ dual mobility cup. The primary outcome was dislocation. Secondary outcomes were revision surgery, 30 days and one year mortality, time to surgery and length of hospital stay.

*Results*: Follow-up time was one year. None of the patients experienced dislocation or received revision surgery in the follow-up period. The 30-days mortality rate was 25% (confidence interval (CI) 95%; 4–46%) and the one year mortality was 45% (CI 95%; 21–69). Mean time to surgery was 27 h (CI 95%; 20–37 h) and mean length of hospital stay was 5.5 days (CI 95%; 4, 0–7, 6 days).

*Conclusion*: THA with the dual-mobility cup seems favourable in the treatment of patients with a displaced femoral neck fracture and patients with dementia. Correct placement of the cup is pivotal and technically demanding. Not all orthopedic surgeons perform total hip arthroplasty while challenges regarding the logistics can be encountered since time to surgery is known to affect the mortality negatively.

## Introduction

Hip arthroplasty is an accepted treatment for displaced femoral neck fractures in elderly patients and is considered to be better than internal fixation [1, 2]. However, hip dislocation after total hip arthroplasty (THA) for femoral neck fractures (FNF) remains a serious complication. Dislocation of THA was the third leading cause of revision between 1979 and 2002 in the Swedish National Hip register [3]. Especially people with dementia are challenging because of difficulties understanding and keeping the necessary hip regimes. Due to a demographic shift toward an older population, a significant growth in numbers of people with dementia and hip fracture is expected over the next decades [4, 5]. It is therefore necessary to identify treatments that can provide a good and stable post-operative clinical result for patients with an acute displaced femoral neck fracture and dementia. This patient group is challenging and has a high risk of complications, morbidity, and mortality after hip fracture [6, 7]. Previous studies have shown 9% dislocation rates for THA and 3% for hemiarthroplasty after acute FNF [8, 9]. However, the dislocation rates may be even higher for patients with dementia, but to our knowledge no studies have evaluated this particular group of patients. The dual mobility acetabular cup (DMC) was introduced with the aim of increasing implant range of motion, stability, and proved secondarily to reduce hip dislocations after THA. In elective patients treated with a THA due to osteoarthritis, the DMC has been associated with decreased dislocation rates [10–15]. The DMC has therefore been suggested as a treatment option in patients with a high risk of hip dislocation, i.e., patients with neuromuscular diseases, cognitive dysfunction, high alcohol intake, high American Society of Anesthesiologist (ASA) score, and patients older than 75 years [10, 16]. To our knowledge no studies have reported the results after THA with the DMC in a selected group of patients with acute FNF and dementia. The primary aim of this study was therefore to describe the dislocation rates after 30 days and one year after THA with the AVANTAGE^®^ dual mobility cup in elderly patients with dementia and displaced FNF. Further, we aimed to describe reoperation rates, mortality 30 day and one year following FNF, time to surgery, and length of hospital stay.

## Materials and methods

From September 2010 to March 2014 we identified 20 patients (18 females, 2 males) median age of 83 years (interquartile range 81–88 years), who were treated with the AVANTAGE^®^ dual mobility cup (Biomet) due to an acute displaced (Garden type 3 or 4) FNF. All patients had a dementia diagnosis and were considered unable to follow the rehabilitation program with restriction of hip flexion and external rotation. Patients were operated at the Regional Hospital Horsens, Denmark which is a teaching hospital covering about 203,000 inhabitants. Annually, approximately 200 hip fracture patients are treated surgically at the hospital.

### Surgical procedure

**Figure 1.**
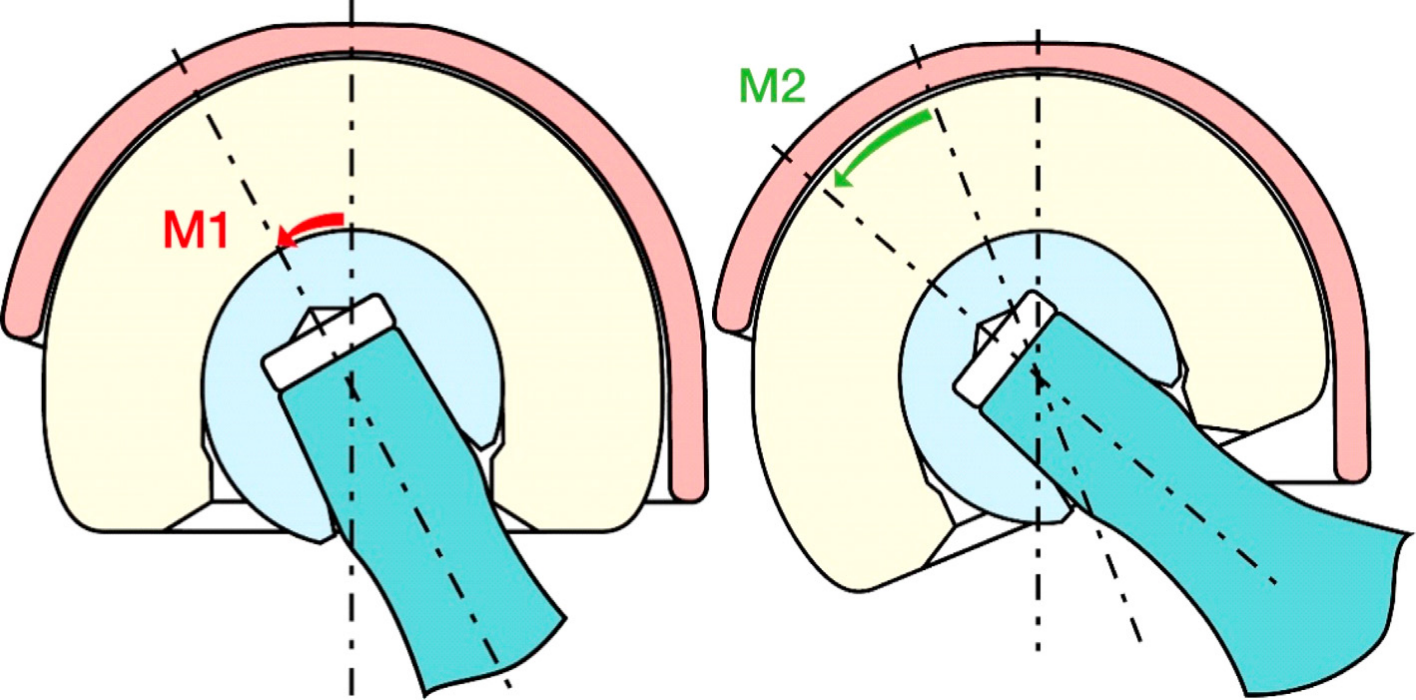
Showing the DMC concept. It consists of two distinct articulations, one between the polyethylene insert and the femoral head (M1) and one between the insert and shell (M2). This allows greater range of motion before impingement of the femoral neck occurs. The figure is the property of Biomet, which have granted their permission for usage in this article.

**Figure 2.**
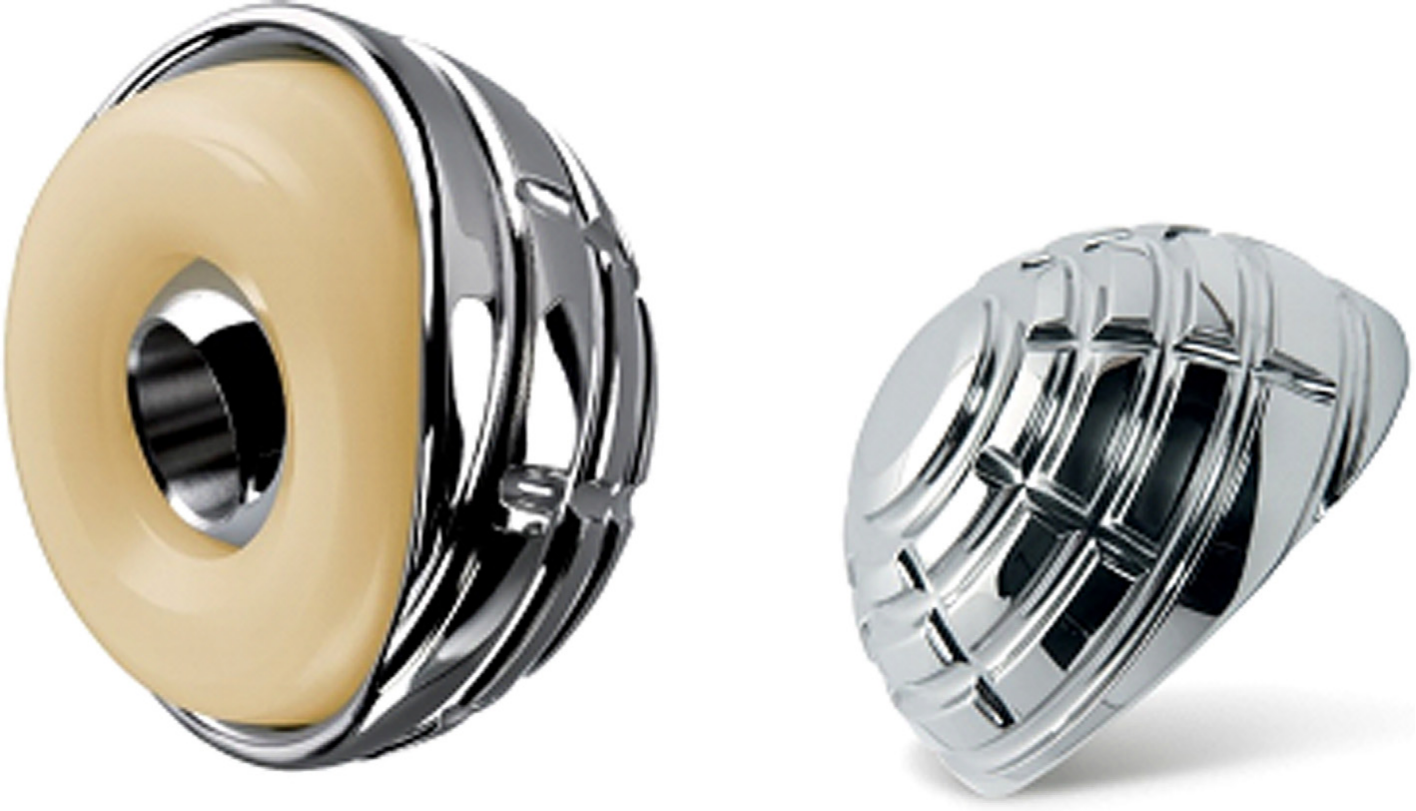
The cemented AVANTAGE^®^ Cup. The 3 mm thick cup cylindro-spherical design is characterized by a superior “cap”. The figure is the property of Biomet, which have granted their permission for usage in this article.

The dual mobility cup offers intra-joint stability through a large diameter mobile liner and large cup coverage. It consists of a combination of two distinct articulations. One constrained articulation between the femoral head and the polyethylene liner, and another unconstrained articulation between the liner and the shell. Flexion occurs mainly in the small inner articulation, and abduction/adduction and rotation starts in the small articulation (Figure 1). This configuration allows for greater range of motion before impingement of the femoral neck occurs and thereby increases implant stability [10, 17]. The cemented AVANTAGE^®^ cup also has an expansion at the posterior rim which makes it even more difficult to dislocate (Figure 2). All procedures, except one cementless, were performed using a cemented AVANTAGE^®^ (Biomet) dual mobility cup (Figure 2) and all except one LUBINUS^®^ stem were treated with an EXETER^®^ stem. Femoral head size was 28 mm when the cup size was ≥50 mm (*n* = 13) and 22.2 mm when the cup size was <50 mm (*n* = 7). With the patients in lateral decubitus the posterior Moore approach with repair of the short external rotators was performed in all patients. Reattachment of the posterior joint capsule (posterior repair) was operator dependent and done in two patients. Patients were allowed immediate weight bearing mobilization the first postoperative day and guided physiotherapy was commenced. All patients were given the same standard instructions concerning motion restriction. Further, all patients received seven days of postoperative thromboprophylaxis with low molecular heparin and antibiotic prophylaxis with IV 750 mg Cefuroxime three times the first postoperative day. Outcome measures and preoperative data were collected by systematic review of the Danish national medical records from March 2015. Preoperative data included age at operation, gender, date of hospitalization, date of operation, and an American Society of Anesthesiologists (ASA) score. Intraoperative data consisted of operating time and surgeon experience divided into consultant and senior resident (Table 1). Furthermore, the cup inclination and the cup position were determined on the postoperative supine antero-posterior and axial cross-table X-rays. We divided the cup position according to Lewinnek et al. [18] in normal (15° anteversion ± 10°), retroversion (<5° anteversion), and anteversion (>25° anteversion) and the inclination in <30°, between 30–50° and >50°, safe zone as between 40° ± 10 (Table 2). The primary outcome was dislocation during a minimum of one-year follow-up period. Secondary outcomes were any additional revision surgery, 30 days and one-year mortality, time to surgery, and length of hospital stay. Time to surgery was defined as time in hours from hospital admission to operation. Length of hospital stay was defined as the time span from hospital admission to hospital discharge or from hip fracture occurrence if the patient was already hospitalized. The discharge date was defined as the date of discharge to home, a nursing home, or death. During the study period senior residents performed 70% of the surgeries and 30% were performed by a consultant.

**Table 1.** Baseline characteristics.

Median age (years)	83 (IQR: 81–88)
Gender (number %)	
Female	18 (90%)
Male	2 (10%)
Cemented	19 (95%)
Uncemened	1 (5%)
Stem	
Exeter	19 (95%)
Lubinus SP-2	1 (5%)
ASA 2	8 (40%)
ASA 3	12 (60%)
Capsular repair	
Yes	2 (10%)
No	18 (90%)
Surgeons	
Consultant	6 (30%)
Senior resident	14 (70%)
Median time of surgery (min)	108 (IQR: 90–123)

ASA = American society of Anesthesiologists; IQR = Interquartile range.

**Table 2.** Cup position.

Cup inclination**	
>50°	4 (24%)
30°–50°	13 (76%)
<30°	0 (0)
Anteversion (+25°)	4 (24%)
Normal (15° ± 10°)	12 (70%)
Retroverted (<5°)	1 (6%)

** Three patients had not pelvis X-ray and could not be assessed.

### Statistical analyses

Time to surgery and length of hospital stay were analyzed as one sample from a normal distribution. We used a natural log transformation to correct for the right skewness in time to surgery and length of hospital stay. The assumption of normality was checked by a QQ plot. Otherwise nonparametric statistic was used, presented as medians with interquartile range (IQR). Mortality, dislocation, and revision were analyzed using exact methods for binomial data. All estimates are given with a 95% confidence interval (95% CI). Stata statistical Software version 12.0 (Stata Corp LP, College Station, Texas, USA) was used for statistical analysis.

## Results

A total of 20 acute FNF treated with the AVANTAGE^®^ dual mobility cup were identified in patients with dementia. The median follow-up time was 12.1 (0.4–47.6) months. Patient characteristics are outlined in Table 1. Eight patients (40%) had an ASA score of 2 and 12 patients (60%) had an ASA score of 3. None of the patients experienced dislocation or received revision surgery in the follow-up period. Five people died within 30 days and the 30-day mortality rate was 25% (95% CI; 4–46%). After one year, nine people had died and the one-year mortality rate was 45% (95% CI; 21–69%) as shown in Table 3. Geometric mean time to surgery was 27 h (95% CI; 20–37 h) and median operative time was 108 min (IQR (90–123 min)). Geometric mean length of hospital stay was 5.5 days (95% CI; 4.0–7.6 days). Mean cup inclination was 48° (95% CI; 47–50°), but four were out of the safe zone with an inclination above 50°. One DMC was retroverted and four were anteverted (Table 2). We did not experience any deep infection.

**Table 3.** Dislocation, revision and mortality.

	30 days 95% CI	1 year 95% CI
Dislocation	None	None
Revision	None	None
Mortality	25% (4–46)	45% (21–69)
Time to surgery (hours)	27 (95% CI; 20–36)	
Length of hospital stay (days)	5.5 (95% CI; 4.0–7.6)	

## Discussion

To our knowledge, this is the first study concerning a highly selected population of patients with dementia treated for an acute displaced femoral neck fracture with a THA and DMC. The results seem promising with respect to dislocation and revision rate after THA with the AVANTAGE^®^ DMC, however the 30-day and one-year mortality rate is high for this group of patients. Even though surgeries were performed by surgeons with variable experience, none of the patients experienced a hip dislocation in the follow-up period. We therefore recommend the DMC in displaced femoral neck fractures in patients with dementia. Two previous studies have used the AVANTAGE^®^ DMC with a good clinical outcome in FNF patients. Tarasevictus et al. [19] compared dislocation rates of the AVANTAGE^®^ DMC with conventional cups in two comparable groups during a period of four years in 98 patients with FNF using the posterior surgical approach. Experienced orthopedic surgeons performed all THAs. At one-year follow-up eight dislocations occurred among 56 conventional THAs whereas no dislocations were reported among the 42 patients treated with a DMC. Four of the eight patients experienced more than one dislocation. None of the dislocations was associated with a significant trauma. However, these patients were not selected as patients with a particularly high risk for dislocation. Similarly, Adam et al. [20] reported three cases of dislocation (1.4%) at nine-month follow-up in a series of 214 randomly selected elderly patients aged 70 and older (mean age 83 years) after treatment for an acute FNF with a DMC. All dislocations occurred between the large articulation of the DMC between the polyethylene liner and the metallic shell. Only one study has reported the results after THA with a DMC in patients with a high risk of dislocation. Sanders et al. [21] reported on 10 hips (eight patients) with cerebral palsy using the AVANTAGE^®^ DMC and they reported no dislocations after a mean follow-up of 39 months.

Another proposed option used to decrease dislocation in high risk patients is the acetabular cups inserted with constrained liners which are now used in THA patients with frequent dislocations. The functional outcome can, however, be negatively affected due to the expected lesser range of motion and theoretically there is a risk of cup loosening or cup dislocation. However, results from Hernigou et al. indicate that constrained liners provided excellent results concerning dislocation rates in neurologic and cognitively impaired patients as well as patients with displaced FNF. Therefore constrained cup liners can be a relevant alternative to both DMC and hemiarthroplasties [22, 23]. In patients with osteoarthritis Combes et al. [24] reported 22 dislocations (0.88%) in 2179 procedures with a DMC after a mean seven years of follow-up. The dislocations occurred between the large articulation in 15 cases whereas seven dislocations occurred between the small articulation. The dislocation between the head and the polyethylene has been of concern because the shell is not designed as a metal-on-metal articulation. This may therefore increase wear dramatically after dislocation. Hemiarthroplasty is a commonly accepted treatment for displaced femoral neck fracture with reported low dislocation rates (3–3.4%) in a general nonselected patient population [9, 25]. You would expect higher dislocation rates for patients with dementia, but currently this information is lacking. The functional outcome and pain after THA has been described as better than hemiarthroplasty [26]. Aseptic loosening and osteolysis has been of concern due to the two articulations in the DMC and a thinner liner which could accelerate polyethylene wear [16, 17, 24]. Concerning our patient population with vulnerable demented elderly people with a low activity level and short remaining time of life, the risk of polyethylene wear and associated aseptic loosening seems of minor importance. For these patients a pain-free joint and a decreased risk of dislocations, revision surgery and readmission seem more important. Knowing the high risk of nosocomial infections, deliria, and mortality during hospitalization of elderly demented patients we find it important to treat the patients with a safe and reliable THA with as few complications as possible. In that context the AVANTAGE^®^ DMC seems to be a good option. The overall 30-day mortality after hip fracture in patients 65 years old and older in a Danish cohort has been reported to be between 10 and 13.2% [27]. For our patient category, the mortality rates were higher, which is expected since our patients have dementia, were relatively old and with higher ASA scores, which are all known predictors for mortality [7]. Comparing our one-year mortality for this specific patient group with van Dortmont et al. [28] our mortality rates are comparable. Another important predictor for mortality is time to surgery [29]. Our surgical delay was 27 h since not all orthopedic surgeons on call perform total hip arthroplasty. Challenges regarding the logistics can be encountered. When interpreting the results of this study, some limitations should be taken into account. The cohort was small and lacking a control group. Further, information concerning the functional outcome as well as patients reported outcome measures would have been of interest for future study. However, these outcomes may not be feasible nor easy to interpret in a cohort of demented patients. In contrast, the external validity of this study seems high as the surgeries were performed by several surgeons with varying experience and we therefore find our results very promising. In conclusion, we recommend the DMC in the treatment of patients with dementia and an acute displaced FNF. At our department DMCs are the first choice if the logistics permits it for patients with dementia and displaced FNF. Correct placement of the cup can be difficult and if not positioned within the safe zones dislocation can occur together with decreased range of motion and soft tissue irritation [18, 20, 30].

## Conflict of interest

Anders Elneff Graversen, Stig Storgaard Jakobsen, Pia Kjær Kristensen, and Theis Muncholm Thillemann declare that they have no conflict of interest.

## References

[1] Rogmark C, Johnell O (2006) Primary arthroplasty is better than internal fixation of displaced femoral neck fractures. A meta-analysis of 14 randomized studies with 2,289 patients. Acta Orthopaedica 77(3), 359–367. 16819672 10.1080/17453670610046262

[2] Hopley C, Stengel D, Ekkernkamp A, Wich M (2010) Primary total hip arthroplasty versus hemiarthroplasty for displaced intracapsular hip fractures in older patients: systematic review. BMJ 340, c2332. 20543010 10.1136/bmj.c2332

[3] The Swedish National Hip Arthroplasty Register (2002) Annual Report. [Online] Available at: http://www.shpr.se/Libraries/Documents/AnnualReport2002-EN.sflb.ashx (accessed 13 June 2015.

[4] Danish Dementia Research Centre (2015) Prevalence of dementia in Denmark. [Online] Available at: http://uk.videnscenterfordemens.dk/dementia-in-denmark/prevalence-of-dementia-in-denmark/ (accessed 11 September 2016).

[5] Gullberg B, Johnell O, Kanis JA (1997) World-wide projections for hip fracture. Osteoporos Int 7, 407–413. 9425497 10.1007/pl00004148

[6] Khan MA, Hossain FS, Ahmed I, Muthukumar N, Mohsen A (2013) Predictors of early mortality after hip fracture surgery. Int Orthop 37, 2119–2124. 23982637 10.1007/s00264-013-2068-1 PMC3824905

[7] Hu F, Jiang C, Shen J, Tang P, Wang Y (2012) Injury preoperative predictors for mortality following hip fracture surgery: a systematic review and meta-analysis. Int J Care Injured 43, 676–685. 10.1016/j.injury.2011.05.017 21683355

[8] Iorio R, Healy WL, Lemos DW, Appleby D, Lucchesi CA, Saleh KJ (2001) Displaced femoral neck fractures in the elderly: outcomes and cost effectiveness. Clin Orthop Relat Res 383, 229–242. 10.1097/00003086-200102000-00027 11210960

[9] Burgers PT, Van Geene AR, Van den Bekerom MP et al. (2012) Total hip arthroplasty versus hemiarthroplasty for displaced femoral neck fractures in the healthy elderly: a meta-analysis and systematic review of randomized trials. Int Orthop 36(8), 1549–1560. 22623062 10.1007/s00264-012-1569-7 PMC3535035

[10] Stroh A, Naziri Q, Johnson AJ, Mont MA (2012) Dual-mobility bearings: a review of the literature. Exp Rev Med Devices 9(1), 23–31. 10.1586/erd.11.57 22145838

[11] Boyer B, Philippot R, Geringer J, Farizon F (2012) Primary total hip arthroplasty with dual mobility socket to prevent dislocation: a 22-year follow-up of 240 hips. Int Orthop 36(3), 511–518. 21698430 10.1007/s00264-011-1289-4 PMC3291786

[12] Guyen O, Pibarot V, Vaz G, Chevillotte C, Béjui-Hugues J (2009) Use of a dual mobility socket to manage total hip arthroplasty instability. Clin Orthop Relat Res 467, 465–472. 18780135 10.1007/s11999-008-0476-0 PMC2628522

[13] Prudhon JL, Ferreira A, Verdier R (2013) Dual mobility cup: dislocation rate and survivorship at ten years of follow-up. Int Orthop 37(12), 2345–2350. 24026216 10.1007/s00264-013-2067-2 PMC3843189

[14] Caton JH, Prudhon JL, Ferreira A, Aslanian T, Verdier R (2014) A comparative and retrospective study of three hundred and twenty primary Charnley type hip replacements with a minimum follow up of ten years to assess whether a dual mobility cup has a decreased dislocation risk. Int Orthop 38(6), 1125–1129. 24737147 10.1007/s00264-014-2313-2 PMC4037498

[15] Prudhon JL (2011) Dual-mobility cup and cemented femoral component: 6 year follow-up results. Hip Int 21(6), 713–717. 22117262 10.5301/HIP.2011.8846

[16] Martino ID, Triantafyllopoulos GK, Sculco PK, Sculco TP (2014) Dual mobility cups in total hip arthroplasty. World J Orthop 5(3), 180–187. 25035820 10.5312/wjo.v5.i3.180 PMC4095010

[17] Grazioli A, Ek ETH, Rüdiger HA (2012) Biomechanical concept and clinical outcome of dual mobility cups. Int Orthop 36(12), 2411–2418. 23073926 10.1007/s00264-012-1678-3 PMC3508052

[18] Lewinnek GE, Lewis JL, Tarr R, Compere CL, Zimmerman JR (1978) Dislocations after total hip-replacement arthroplasties. J Bone Joint Surg Am 60, 217–220. 641088

[19] Tarasevicius S, Busevicius M, Robertsson O, Wingstrand H (2010) Dual mobility cup reduces dislocation rate after arthroplasty for femoral neck fracture. BMC Musculoskelet Disord 11, 175. 20691056 10.1186/1471-2474-11-175 PMC2922087

[20] Adam P, Philippe R, Ehlinger M et al. (2012) Dual mobility cups hip arthroplasty as a treatment for displaced fracture of the femoral neck in the elderly. A prospective, systematic, multicenter study with specific focus on postoperative dislocation. Orthop Traumatol Surg Res 98, 296–300. 22463868 10.1016/j.otsr.2012.01.005

[21] Sanders RJ, Swierstra BA, Goosen JH (2013) The use of a dual-mobility concept in total hip arthroplasty patients with spastic disorders: no dislocations in a series of ten cases at midterm follow-up. Arch Orthop Trauma Surg 133, 1011–1016. 23632783 10.1007/s00402-013-1759-9

[22] Hernigou P, Filippini P, Flouzat-Lachaniette CH, Batista SU, Poignard A (2010) Constrained Liner in neurologic or cognitively impaired patients undergoing primary THA. Clin Orthop Relat Res 468, 3255–3262. 20376709 10.1007/s11999-010-1340-6 PMC2974891

[23] Hernigou P, Ratte L, Roubineau F, Pariat J, Mirouse G, Guissou I, Allain J, Lachaniette CHF (2013) The risk of dislocation after total hip arthroplasty for fractures is decreased with retentive cups. Int Orthop 37, 1219–1223. 23665654 10.1007/s00264-013-1911-8 PMC3685674

[24] Combes A, Migaud H, Girard J, Duhamel A, Fessy MH (2013) Low rate of dislocation of dual-mobility cups in primary total hip arthroplast. Clin Orthop Relat Res 471, 3891–3900. 23516032 10.1007/s11999-013-2929-3 PMC3825881

[25] Varley J, Parker MJ (2004) Stability of hip hemiarthroplasties. Int Orthop 28, 274–277. 15316673 10.1007/s00264-004-0572-z PMC3456984

[26] Macaulay W, Nellans KW, Garvin KL, Iorio R, Healy WL, Rosenwasser MP (2008) Prospective randomized clinical trial comparing hemiarthroplasty to total hip arthroplasty in the treatment of displaced femoral neck fractures: winner of the Dorr Award. J Arthroplasty 23(6 Suppl 1), 2–8. 10.1016/j.arth.2008.05.013 18722297

[27] Kristensen PK, Thillemann TM, Johnsen SP (2014) Is bigger always better? A nationwide study of hip fracture unit volume, 30-day mortality, quality of in-hospital care, and length of hospital stay. Med Care 52(12), 1023–1029. 25226544 10.1097/MLR.0000000000000234

[28] van Dortmont LM, Douw CM, van Breukelen AM et al. (2000) Outcome after hemi-arthroplasty for displaced intracapsular femoral neck fracture related to mental state. Injury 31(5), 327–331. 10775686 10.1016/s0020-1383(99)00304-6

[29] Daugaard CL, Jørgensen HL, Riis T, Lauritzen JB, Duus BR, van der Mark S (2012) Is mortality after hip fracture associated with surgical delay or admission during weekends and public holidays? A retrospective study of 38,020 patients. Acta Orthopaedica 83(6), 609–613. 23140106 10.3109/17453674.2012.747926 PMC3555458

[30] Mukka SS, Mahmood SS, Sjödén GO, Sayed-Noor AS (2013) Dual mobility cups for preventing early hip arthroplasty dislocation in patients at risk: experience in a county hospital. Orthop Rev 5(2), 48–51. 10.4081/or.2013.e10 PMC3718234 23888200

